# Expression and Regulation of 11-****β**** Hydroxysteroid Dehydrogenase Type 2 Enzyme Activity in the Glucocorticoid-Sensitive CEM-C7 Human Leukemic Cell Line

**DOI:** 10.1155/2013/245246

**Published:** 2013-03-17

**Authors:** Mark R. Garbrecht, Thomas J. Schmidt

**Affiliations:** ^1^Department of Biology, Winona State University, 234 Pasteur Hall, P.O. Box 5838, Winona, MN 55987, USA; ^2^Department of Molecular Physiology and Biophysics, Roy J. and Lucille A. Carver College of Medicine, University of Iowa, 6-452 Bowen Science Building, 51 Newton Road, Iowa City, IA 52242, USA

## Abstract

Glucocorticoids are commonly used in the first-line treatment of hematological malignancies, such as acute lymphoblastic leukemia, due to the ability of these steroids to activate pro-apoptotic pathways in human lymphocytes. The goal of the current study was to examine the gene expression and enzyme activity of the microsomal enzyme, 11-**β** hydroxysteroid dehydrogenase type 2 (HSD11B2, HSD2), which is responsible for the oxidation of bioactive glucocorticoids to their inert metabolites. Using the glucocorticoid-sensitive human leukemic cell line, CEM-C7, we were able to detect the expression of HSD2 at the level of mRNA (via RT-PCR), protein (via immunohistochemistry and immunoblotting), and enzyme activity (via conversion of tritiated cortisol to cortisone). Furthermore, we observed that HSD2 enzyme activity is down regulated in CEM-C7 cells that were pretreated with the synthetic glucocorticoid, dexamethasone (100 nM, <15 hours), and that this down regulation of enzyme activity is blocked by the administration of the glucocorticoid receptor antagonist, RU-486. Taken collectively, these data raise the possibility that the effectiveness of glucocorticoids in the treatment of human leukemias may be influenced by: (1) the ability of these neoplastic cells to metabolize glucocorticoids via HSD2 and (2) the ability of these steroids to regulate the expression of this key enzyme.

## 1. Introduction

Glucocorticoid (GC) therapy remains one of the first-line therapies in the treatment of a variety of leukemias, including acute lymphoblastic leukemia (ALL). Synthetic GCs, such as dexamethasone and prednisone, are effective in treating ALL due to their ability to induce cell cycle arrest and apoptosis. 

The microsomal enzyme, 11-beta hydroxysteroid dehydrogenase type 2 (HSD2), catalyzes the conversion of biologically active 11-hydroxy glucocorticoids (i.e., cortisol) to their inactive 11-keto metabolites (i.e., cortisone). This activity allows for the tissue-specific conversion of cortisol to inactive cortisone, thereby decreasing the local concentrations of active GCs. Loss of the ability to inactivate cortisol via HSD2 enzyme activity has been implicated in the pathogenesis of a number of human diseases [1].

Despite the widespread use of GCs to treat leukemia, relatively little is known about the metabolism of GCs in human leukemic cells. The CEM-C7 cell line is glucocorticoid-sensitive subclone of a human leukemic cell line derived from a 4-year-old female patient with late stage ALL [2, 3]. A previous study has reported that the synthetic GC, dexamethasone, was not metabolized to an appreciable degree by CEM-C7 cells, concluding at that time that peripheral metabolism likely did not play a role in the response of leukemic cells to glucocorticoids [4]. Since that time, a number of studies have shown that dexamethasone is a poor substrate for HSD2 [5]. As a result, the presence and/or significance of HSD2 may have been overlooked in this glucocorticoid-sensitive subclone. The goal of this paper was to examine the expression of the HSD2 enzyme and the metabolism of cortisol in CEM-C7 cells.

## 2. Methods

### 2.1. Cell Culture

Glucocorticoid-sensitive CEM-C7 cells were grown in suspension in RPMI 1640 medium supplemented with 10% fetal bovine serum (FBS), 1% antibiotic/antimycotic solution, and 2 mM L-glutamine (Invitrogen, Carlsbad, CA) at 37°C in a humidified atmosphere of 95% air/5% CO_2_. Media were changed biweekly, and the cells were passed weekly, maintaining cell densities of 5 × 10^4^ to 1 × 10^6^ per milliliter. Cells were serum-starved for 24 hours prior to treatments to avoid the influence of endogenous GCs found in FBS. For experiments, the cells were incubated in serum-free media containing either vehicle (0.0001% ethanol), 100 nM dexamethasone (Dex), or Dex plus RU-486 (2 *μ*M). Steroids were purchased from Sigma (St. Louis, MO), and 1 mM stock solutions were prepared in ethanol. 

### 2.2. Immunohistochemistry

CEM-C7 cells were spotted and grown in serum-containing culture medium on chambered glass Lab-Tek microscope slides (Thermo Scientific, Rochester, NY) for 48 hours prior to immunostaining. Cells were fixed in formalin (10% formaldehyde in phosphate buffered saline) and immunostained as previously described using a Vectastain Elite ABC kit (Vector Laboratories, Burlingame, CA) and HSD2-specific antibody at a concentration of 200 ng/mL, or secondary antibody alone for negative controls [6].

### 2.3. RNA Isolation

Total RNA was isolated from CEM-C7 cells using TRIzol reagent (Invitrogen, Carlsbad, CA) according to the manufacturer's directions. The RNA was resuspended in ultrapure water and quantified by determining the absorbance at 260 nM. RNA integrity was monitored by examining the 28S and 18S rRNA bands after electrophoresis in agarose gels stained with ethidium bromide.

### 2.4. Reverse Transcription Polymerase Chain Reaction (RT-PCR)

One microgram of total RNA was reverse transcribed using oligo-dT 18 mers as primers in a reaction volume of 30 *μ*L that contained dNTP mix (2.5 mM each), 5 units of mouse murine leukemia virus (MMLV) reverse transcriptase, and the supplied reaction buffer (Invitrogen, Carlsbad, CA). The RT reaction was carried out at 37°C for 1 hour. Five microliters of the resulting cDNAs or 5 *μ*L of water (negative control) was used as template in PCR reactions using human HSD2-specific primers that span multiple exons (forward: 5′ CGGCTGTGACTCTGGTTTTGGCAA 3′, reverse: 5′ ATAGGCCCCCAAGCACGGATAT 3′). Amplification was performed for 35 cycles (95°C denaturation for 30 seconds, 54°C annealing for 90 seconds, and 72°C extension for 120 seconds) followed by a final extension cycle of 10 minutes at 72°C. PCR products were resolved on a 2% agarose gel containing ethidium bromide. Purified PCR products were sequenced by the DNA Core Facility of the University of Iowa and compared to sequence data published in GenBank.

### 2.5. HSD2 Enzyme Activity Assays

Control and treated CEM-C7 cells grown to approximately 80% confluency in 100 mm culture plates were washed with phosphate buffered saline and then incubated in serum-free media containing 1,2-[^3^H]-cortisol (41 Ci/mmol, American Radiolabeled Chemicals, St. Louis, MO) at a final concentration of 1 nM for 24 hours at 37°C. Following incubation, the CEM-C7 cells were harvested by centrifugation for 10 minutes at 1,000 ×g. The labeled steroids were extracted from the media by adding 2 volumes of ethyl acetate and vortexing for 1 minute followed by centrifugation for 10 minutes at 600 ×g. The ethyl acetate phase was dried under N_2_ gas and the steroids resuspended in ethanol. The resuspended steroids were spotted onto silica-coated glass plates (silica gel G, Analtech, Newark, DE) and resolved in an equilibrated thin-layer chromatography (TLC) chamber using chloroform/ethanol (92 : 8) as the solvent. [^3^H]-Steroids were detected and quantified using a TLC plate reader (Bio Scan AR-2000, Bio Scan Inc., Washington, DC) capable of detecting tritium. HSD2 oxidase activity was assessed by calculating the conversion of [^3^H]-cortisol to [^3^H]-cortisone. Parallel assays were performed in flasks containing no cells to monitor for spontaneous interconversion between 11-hydroxy- and 11-dehydroforms of the steroids. Percent conversion values were normalized to protein content total protein homogenates from corresponding CEM-C7 cell pellets. 

### 2.6. Protein Isolation and Western Blot Analysis

Total homogenate protein was isolated by homogenization of CEM-C7 cells, adult human liver tissue, or adult human kidney tissue (BioChain Institute, Hayward, CA) in lysis buffer (1 nM phenylmethylsulfonyl fluoride in PBS), followed by centrifugation (10 minutes at 600 ×g) and isolation of the supernatant. Protein concentration of the lysates was quantified using Bio-Rad protein assay reagent (Bio-Rad, Hercules, CA). One hundred micrograms of homogenate protein from CEM-C7 cells or fifty micrograms of human kidney or liver homogenate protein was separated via one-dimensional sodium dodecyl sulfate polyacrylamide gel electrophoresis (SDS-PAGE) under denaturing conditions and subsequently transferred to nitrocellulose membrane. Immunoblotting was performed using a human HSD2-specific primary antibody (HUH23) at a concentration of 2 *μ*g/mL as previously described [7]. Homogenate proteins of human liver and kidney (BioChain, Hayward, CA) were used as negative and positive controls for the expression of HSD2, respectively.

## 3. Results

### 3.1. Expression of HSD2 in the CEM-C7 Cell Line

**Figure 1:**
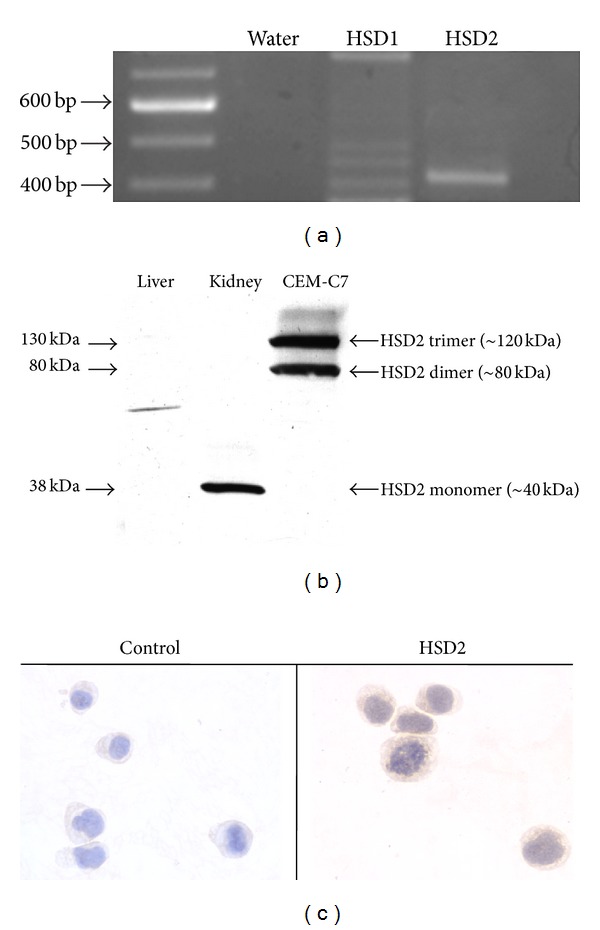
Expression of HSD2 in CEM-C7 cells. (a) mRNA for HSD2 was detected by RT-PCR analysis of total mRNA isolated from CEM-C7 cells. (b) Immunoreactive HSD2 protein was detected in homogenates of CEM-C7 cells, and human kidney was included as a positive control. (c) Cellular expression of HSD2 was also detected via immunohistochemistry.

In order to assess the expression of the HSD1 and HSD2 genes, reverse transcription PCR (RT-PCR) was performed on total mRNA isolated from CEM-C7 cells using PCR primers specific for the HSD1 and HSD2 mRNAs. As shown in Figure 1(a), the CEM-C7 cell line expresses the mRNA for HSD2, but not HSD1. Once the expression of the HSD2 gene was confirmed by sequencing of the cDNA transcript, we sought to establish the expression of the HSD2 protein and demonstrate functional HSD2 enzymatic activity. As shown in Figure 1(b), HSD2 protein was detected in CEM-C7 cells via immunoblot analysis as a dimer and trimer of approximately 80 and 120 kDa. Cytoplasmic expression of the HSD2 protein was further confirmed via immunohistochemistry as shown in Figure 1(c), consistent with its localization to the endoplasmic reticulum. 

### 3.2. HSD2 Activity and Regulation by Dexamethasone

**Figure 2:**
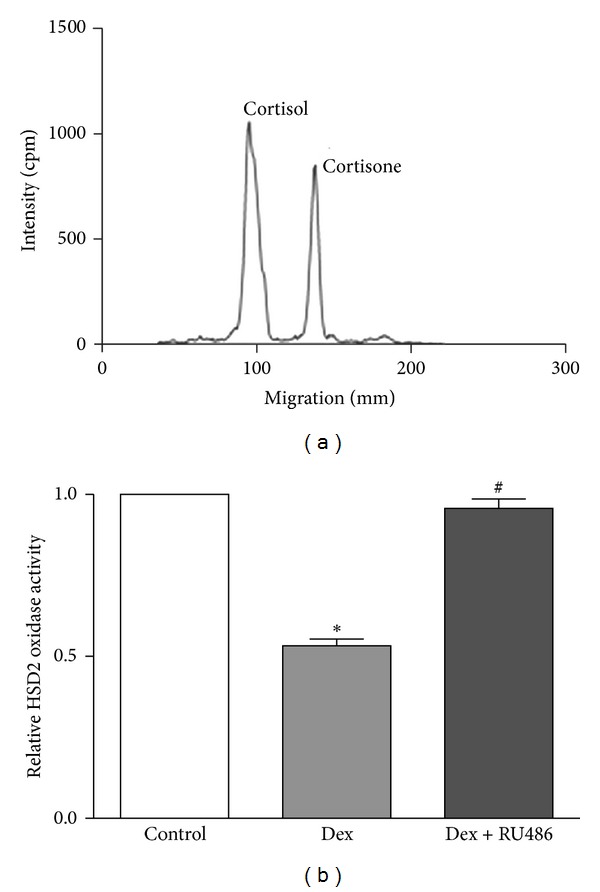
HSD2 enzyme activity in CEM-C7 cells and its regulation by Dex. (a) Intact CEM-C7 cells actively metabolized ^3^H-labeled cortisol to cortisone, indicative of HSD2-specific enzyme activity. Pretreatment of CEM-C7 cells with 100 nM Dex reduced HSD2-specific enzyme activity. (b) The Dex-mediated reduction in HSD2 activity was blocked by 2 *μ*M RU486. *n* = 4 independent experiments; *P ≤ 0.05 as compared to controls and ^#^ *P* ≤ 0.05 as compared to Dex treated cells via Student's *t*-test.

In order to confirm the functional activity of the HSD2 gene in CEM-C7 cells, the metabolism of radioactively labeled cortisol was examined in cultured, intact cells. As shown in Figure 2(a), CEM-C7 cells actively catalyzed the oxidation of biologically active cortisol to inactive cortisone. In our experiments, we observed average conversion rates of cortisol to cortisone of 24.93 ± 2.08%. Since the HSD2 gene had previously been shown to be regulated by GCs, we also examined the effects of glucocorticoid receptor (GR) stimulation on HSD2 activity in the CEM-C7 cell line [7–9]. As shown in Figure 2(b), an overnight (≤15 hours) pretreatment of CEM-C7 cells with 100 nM Dex resulted in a significant decrease (53.5 ± 2.07% of control) in HSD2 enzyme activity in these cells. Furthermore, we observed that this effect was blocked by coincubation with a 20-fold molar excess of the GR antagonist, RU-486 (95.7 ± 2.9% of control). 

## 4. Discussion

In this paper, we have shown that a glucocorticoid-sensitive subclone of the CCRF-CEM cell line, CEM-C7, expresses the mRNA and protein for the GC metabolizing enzyme, HSD2. We have further shown that these cells also possess functional HSD2 enzyme activity, as demonstrated by the ability of these cells to oxidize biologically active cortisol to cortisone. In addition, it appears that the activity, and perhaps gene expression, of HSD2 is regulated by GR expressed in these cells. This is demonstrated by the finding that HSD2 enzyme activity is downregulated in cells that are pretreated for <15 hours and that this downregulation is completely blocked by the GR antagonist, RU-486. The effect of RU486 demonstrates that the regulation of HSD2 activity by Dex is a receptor-mediated response rather than a potential allosteric effect caused by direct interaction between the enzyme and the GR agonist (Dex).

As shown in Figure 1(b), we were able to detect the expression of the HSD2 protein in the dimer (~80 kDa) and trimer (~120 kDa) forms from CEM-C7 cell total protein homogenates. However, despite the use of denaturing reagents (sodium dodecyl sulfate, 2-mercaptoethanol), we were not able to detect immunoreactive HSD2 protein in the ~40 kDa monomer form as is clearly seen in the positive control (human kidney). No immunoreactive bands corresponding to HSD2 (monomer, dimer, or trimer) were detected in our negative control of human liver homogenate. In our experiments, as well as those conducted by others (Z. Krozowski, unpublished observations), the HSD2-specific antibody (HUH23) utilized in this study variably yields HSD2-specific bands that correlate to monomers, dimers, and trimers. Using this antibody, we have previously detected these bands in cultured lung epithelial cells (NCI-H441), cultured colon epithelial cells (CaCo-2), and in human adult and fetal lung tissues [7, 10]. Furthermore, HSD2 has been previously shown to exist in dimer form under oxidizing conditions [11]. Additionally, we have observed that in H441 cells, CaCo-2 cells, and adult lung tissue, the HSD2 monomer is the least abundant of the three multimeric forms, suggesting the possibility that in CEM-C7 cell homogenates the monomer form may have been below the level of immunodetection under our assay conditions, despite our clear demonstration of HSD2 mRNA (Figure 1(a)) and functional HSD2 enzymatic activity (Figure 2(a)). 

In addition to observing the functional activity of the HSD2 enzyme in CEM-C7 cells, we also observed that the GR agonist, Dex, downregulates the amount of HSD2 enzyme activity present in these cells. For our studies, we chose to use Dex, since it is known to be a poor substrate for oxidative metabolism by HSD2 (thereby limiting its breakdown following administration) and also since Dex retains significant GR agonist activity in its 11-keto form [5, 7]. As shown in Figure 2(b), we observed a downregulation of HSD2 enzyme activity in cells that were pretreated with 100 nM Dex and that this effect was blocked by the GR antagonist, RU-486. The regulation of HSD2 activity by Dex, and abrogation of this effect by RU-486, has previously been observed in other cell types, including placental trophoblast cells [12]. In our studies, CEM-C7 cells were treated with 100 nM Dex for 12 to 15 hours, which is a concentration and time frame that have been previously shown to have no detectable effect on cell viability [13]. Furthermore, the expression of another enzyme, glutamine synthetase, has been reported to be upregulated in these cells following a 24-hour treatment with a concentration of Dex that was 10-fold higher than that used in the current study [14]. This result suggests that under our experimental conditions, Dex does not simply downregulate all enzymes expressed in CEM-C7 cells. Thus, Dex appears to act via the GR to downregulate HSD2 activity by one or more potential mechanisms such as (1) downregulation of HSD2 gene transcription, (2) a posttranscriptional reduction in the half-life of the HSD2 mRNA, or (3) a post-translational modification resulting in a reduction of the half-life of the HSD2 protein. Further studies will be required to elucidate the exact mechanism by which Dex influences HSD2 activity in CEM-C7 cells. 

To our knowledge, this paper represents the first report of HSD2 gene and enzyme activity expression in the CEM-C7 cell line. One recent study by Sai et al. demonstrated that the protein and mRNA expression of HSD2 was negligible in the GC-sensitive CCRF-CEM cell line but was more abundant in another human leukemic cell line that is GC resistant (MOLT4F cells) [15]. The CCRF-CEM cell line is an uncloned human leukemic cell line that exhibits partial GC sensitivity and is the cell line from which the CEM-C7 cell line was cloned [3]. Therefore, the CEM-C7 cell line may represent a unique subclone that expresses higher levels of HSD2 than the uncloned, parental cell line, which highlights the possible heterogeneous nature of HSD2 expression in human leukemic cells. Finally, since GCs play a key role in the combined chemotherapy for the treatment acute lymphoblastic leukemia, a better understanding of the metabolism of these steroids by leukemic cells will offer additional information on the biology of these cells and potentially provide insight in the future treatment of GC-resistant leukemias.

## 5. Conclusions

In summary, this paper presents the first data concerning the expression of the HSD2 gene in the glucocorticoid-sensitive CEM-C7 human leukemic cell line. In addition to confirming the expression of HSD2 mRNA, we have also demonstrated the expression of HSD2 protein, its cellular localization, and the functional activity of the HSD2 enzyme as evidenced by the conversion of cortisol to cortisone. We also demonstrate that the glucocorticoid receptor agonist, Dex, influences the activity of the HSD2 enzyme in these cells, likely in a GR-dependent manner as this downregulation is blocked by the GR antagonist, RU-486. This, and future work, is aimed at elucidating the potential role of glucocorticoid-metabolizing enzymes in human leukemic cells and how these cells metabolize different endogenous and synthetic glucocorticoid hormones.
